# Tropospheric Ozone Formation Estimation in Urban City, Bangi, Using Artificial Neural Network (ANN)

**DOI:** 10.1155/2019/6252983

**Published:** 2019-05-23

**Authors:** Fatin Aqilah Binti Abdul Aziz, Norliza Abd. Rahman, Jarinah Mohd Ali

**Affiliations:** ^1^Chemical Engineering Programme, Faculty of Engineering and Built Environment, Universiti Kebangsaan Malaysia, UKM Bangi, 43600 Selangor, Malaysia; ^2^Research Centre for Sustainable Process Technology, Faculty of Engineering and Built Environment, Universiti Kebangsaan Malaysia, UKM Bangi, 43600 Selangor, Malaysia

## Abstract

Due to the rapid development of economy and society around the world, the most urban city is experiencing tropospheric ozone or commonly known as ground-level air pollutants. The concentration of air pollutants must be identified as an early precaution step by the local environmental or health agencies. This work aims to apply the artificial neural network (ANN) in estimating the ozone concentration forecast in Bangi. It consists of input variables such as temperature, relative humidity, concentration of nitrogen dioxide, time, UVA and UVB rays obtained from routine monitoring, and data recorded. Ten hidden layer is utilized to obtain the optimized ozone concentration, which is the output layer of the ANN framework. The finding showed that the meteorology condition and emission patterns play an important part in influencing the ozone concentration. However, a single network is sufficient enough to estimate the concentration despite any circumstances. Thus, it can be concluded that ANN is able to give reliable and satisfactory estimations of ozone concentration for the following day.

## 1. Introduction

Clean air is a necessity for human wellbeing and prosperity. Air contamination is a genuine danger to both human and the Earth; therefore, step-by-step modern exercises are practiced to accomplish the globalization, which has been influencing the surrounding air and deterioration of human's condition. Tropospheric ozone (O_3_) is a worldwide air contamination and harming substance issues. Despite the fact that no major issue has been reported, ground-level ozone remains as the most unavoidable air poisons worldwide, which will affect human wellbeing, generation sustainability, and the Earth [1]. Ozone can affect health, especially lead to breathe problem, inflame and damage the airways, and deteriorate lung function in both children and adults, emphysema, and asthma attacks [2].

Ozone is a basic environmental element that exists in the stratosphere and troposphere of the Earth. Stratospheric ozone incorporates bright beams in the broad daylight and prevents them from reaching the Earth surface. The bright lights obstructed by stratospheric ozone are unsafe for living things, and it is known as “great ozone.” On the other hand, ozone is an auxiliary contamination produced in the troposphere when nitrogen oxides (NO_*x*_) and volatile organic compound (VOC) respond during the day 1. Nevertheless, tropospheric ozone ingests infrared beams radiating from the Earth and acts as an effective ozone-depleting substance. As indicated by the Intergovernmental Board on Environmental Change [3], tropospheric ozone is considered as the third most effective ozone harming substance, after carbon dioxide and methane. At the ground level, it is a significant air poison and one of the primary oxidants. Due to its nursery impact and poisonous quality, tropospheric ozone is known as “awful ozone” [4].

The imminence of ozone towards the environment will affect human with intense consequences to the respiratory framework, interminable infection, and mortality. These respiratory failures will lead to decreasing of lungs' capacity, lung aggravation, and irritation of the respiratory tract [5]. In the EU, approximately 21,400 unexpected losses that were related to O_3_ have been recorded yearly [6]. Ozone has been identified as harmful to delicate plant species, able to decrease tree development, and may influence the group structure of normal plants. Thus, it has clear implications on future security sustainability [1].

Current researches have shown that ANN-based air pollution models perform better than other statistical techniques [7, 8]. ANN is one of the artificial intelligence elements, which is developed based on similar terminology of human brain process information [9, 10]. Applications of ANN in atmospheric sciences have begun in the late 90s and have been proven to succeeding in forecasting model [11, 12]. The ANN technique has been proven to be a success estimator [13] since it is statistically able to learn complex relationships between inputs and outputs. The objective of this work is to apply ANN to estimate the ozone concentration based on the data obtained in Bangi. Congestion on road in Bandar Baru, Bangi, where all vehicles emitted the smog containing other chemicals such as NO_*x*_ and VOCs is the factor contributing to the formation of tropospheric ozone.

Depletion of stratospheric ozone allows the UVB rays to penetrate the ozone layer, thus increases the photolysis reaction of the ozone. Besides that, this research aims to study the relationship between the concentration of ozone in congested area in Bangi and its relation with the buildup of nitrogen dioxide and the relationship between the parameter towards the formation of ozone. ANN model predictor will also aid to forecast the air pollution when the major factor that form the ozone in the tropospheric layer has been identified during the estimation process. This chapter is the introduction followed by the methodology in Section 2. The results are given in Section 3 with the finding discussion. Finally, the conclusion is given in Section 4.

## 2. Methodology

In this paper, the ozone (O_3_) concentration, nitrogen dioxide (NO_2_), UV intensity, wind speed and direction, temperature, and humidity data are collected and analyzed. Based on the data, a neural network framework is then developed using MATLAB toolbox for forecasting of the daily basis pollution [17]. The toolbox has aided in the estimation and data analysis.

Ozone formation is driven by two transmitted antecedents, namely, nitrogen oxides (NO_*x*_) and volatile organic compound (VOC). NO_*x*_ is a term used to describe mononitrogen oxides (NO) and nitrogen dioxides (NO_2_). VOCs are a mix of chemicals discharged from both common and man-made sources. Characteristic sources incorporate petroleum derivative stores, volcanic outflows, and uncontrollable fires. Human activities will influence the establishment of VOCs such as discharge from engine vehicle, pesticides, and gas vapors. These mixtures are trapped from the response of nitrogen and oxygen at hoisted temperature. Ozone is framed from the response of NO_*x*_ with unpredictable natural compound within the sight of UV beams from daylight [7]. Furthermore, if the temperature is high, the rate of reactions will increase, thus increase the ozone concentrations. The chemical reaction involves in the formation of ozone in the atmosphere, given as [8]

(1) $$\begin{array}{c} {\text{O}}_{2}+hv⟶\text{O}+\text{O}\;\left(\lambda <242\text{ nm}\right)\\ \text{O}+{\text{O}}_{2}⟶{\text{O}}_{3}\\ {\text{O}}_{3}+hv⟶\text{O}+{\text{O}}_{2}\\ \text{O}+{\text{O}}_{3}⟶{\text{O}}_{2}+{\text{O}}_{2} \end{array}$$

Formation of ozone occurs through the following sequence of reactions. First, it is always initiated by reactions of various VOC or CO with the OH radical. NO_2_ is photolyzed to generate atomic oxygen which then combines with oxygen (O_2_) to create ozone (O_3_). The rate of ozone formation is controlled primarily by the rate of the initial reaction of VOC with OH. The following equation is the sequence of chemical reactions of ozone formation involving VOC and NO_*x*_ [14]:

(2) $$\begin{array}{c} \text{VOC}+\text{OH}⟶{\text{RO}}_{2}+{\text{H}}_{2}\text{O}\\ \text{CO}+\text{OH}⟶{\text{HO}}_{2}+{\text{CO}}_{2}\\ {\text{RO}}_{2}+\text{NO}⟶\text{VOC}+{\text{H}}_{2}\text{O}+{\text{NO}}_{2}\\ {\text{HO}}_{2}+\text{NO}⟶\text{OH}+{\text{NO}}_{2}\\ {\text{NO}}_{2}+hv⟶\text{NO}+\text{O}\\ \text{O}+{\text{O}}_{2}+\text{M}⟶{\text{O}}_{3}+\text{M} \end{array}$$

### 2.1. Data Collection

**Figure 1:**
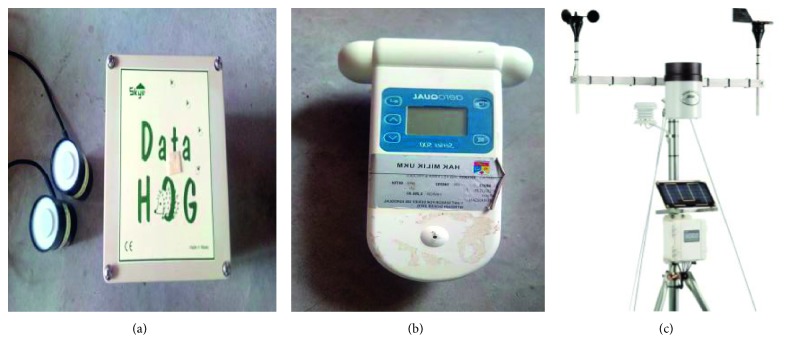
Equipment to run the experiment.

The equipment used to obtain the concentration of ozone (O_3_) is addressed as Aeroqual series 500 portable ozone monitor. This portable monitor will be left near the experimental area within 7 hours throughout the experiment. It is basically carried out for seven random days in between February 2018 and April 2018. All the recorded data will be kept in the data logger before transferring to the computer for further analysis. On the other hand, the *Aeroqual series 500 portable gas sensor* is applied to collect the nitrogen dioxide (NO_2_)*, SKYE Instrument MiniMet weather station* is used to capture the UV light, and *HOBO U30 weather station kit* is employed for surrounding weather data accumulation. For the UV light collection, the equipment is equipped with UVA and UVB sensors and DataHog to record the data. The experiments are conducted with the assistance of monitoring and recording device. The devices have been calibrated before starting the experiment. From manual, the user only has to calibrate if the devices have not been used for a long time [18]. Thus, all devices have been correctly calibrated, and the readings are acceptable. The equipment is illustrated in Figure 1.

### 2.2. Data Analysis

The Statistical Packages for the Social Science (SPSS) is widely used software for statistical analysis in social sciences. This product is known as a statistical package that can process complex manipulation data and analyze using simple instructions. It is designed for both interactive and noninteractive use. Here, we applied the IBM SPSS software for analyzing the data, which offers an advanced statistical analysis, extensive algorithms, text analysis, open-source involvement, and complex data integration. In analyzing the data from each parameter, we utilize Pearson correlation to observe the relationship between each parameter, and a simple linear regression will help to explain the regression between the concentration of ozone to independent parameter such as concentration of nitrogen dioxide and UV intensity.

### 2.3. Artificial Neural Network Framework

**Figure 2:**
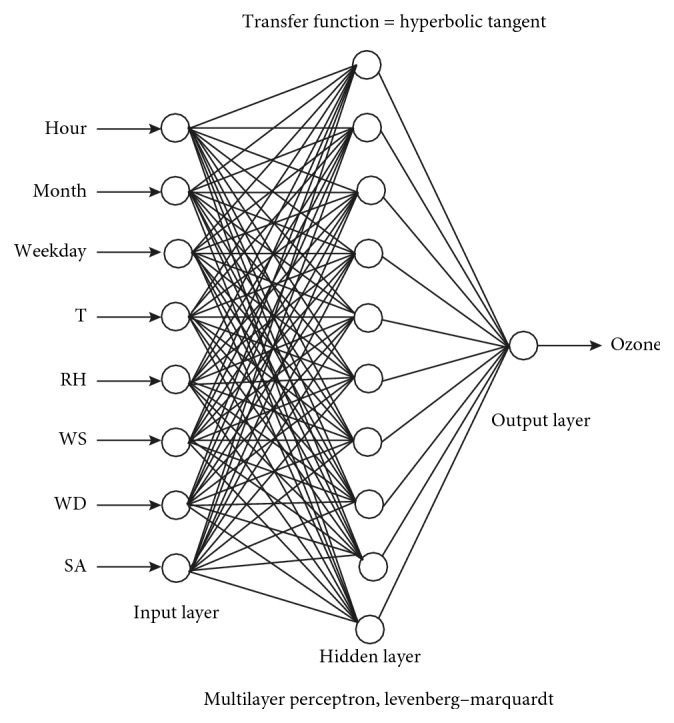
The neural network framework.

Based on the data collection, a neural network framework is developed using a multilayer feed-forward back propagation network, which comprises of an input layer, a hidden layer, and an output layer. All data in the input layer will be feed-forwarded to the hidden layer as the next layer. Levenberg–Marquardt algorithm is chosen as the learning algorithm with 43% adequacy. This framework is designed in MATLAB software using the data from the third day of the air pollution occurrence. Day three is chosen since the data showed a stable pattern compared to other days. The framework is given in Figure 2, and the detail information is tabulated in Table 1.

The ANN model has been trained and genetically optimized. The optimized mean square error (MSE) shows a value less than 0.01. The developed ANN models have been assessed by comparing its predicted output results, which is the forecasted pollution on the next day in this case through network training, validation, and data sets testing, respectively [15]. Regression value that varies from 0 to 100 percent will represent the performance of the model. Furthermore, forward stepwise selection has identified that the concentration of nitrogen dioxide gave the significance effect. The results of the optimized network with all inputs of the experimental results versus the predicted output are plotted and analyzed.

## 3. Results and Discussion

**Figure 3:**
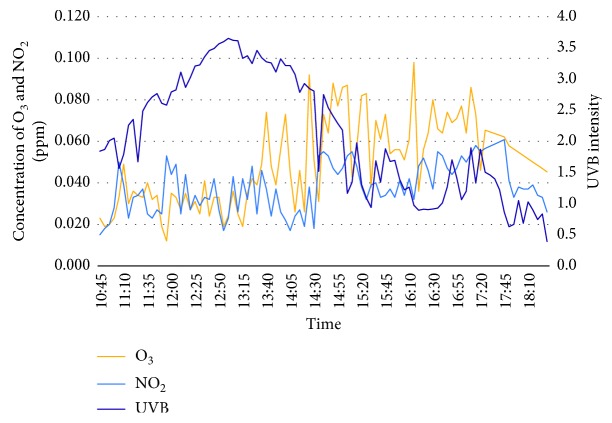
Concentration of O_3_, NO_2_, and UVB Intensity for day 1 (20^th^ Feb 2018).

The first day of the experiment is on the 20^th^ February 2018 whereby the surrounding weather is more than 34°C in the middle of the day (12 to 3 pm) as well as during peak hours (5 to 6 pm). Both the concentration of ozone and nitrogen dioxide has been observed to be in a moderate concentration from early morning till afternoon, but is slowly increasing towards peak hours in the evening. This is resulted from passing vehicles accumulating the road to exit or enter Bangi. In addition, UVB intensity has shown a negative regression where the highest intensity was observed during midday and slowly decreases from late afternoon till evening. It was a bright sunny day throughout the experiment, and cloud can be seen covering the sun radiation, which lead to the high value of UVB intensity.  Figure 3 shows the graph of concentration of ozone, nitrogen dioxide, and UVB intensity for day 1.

**Figure 4:**
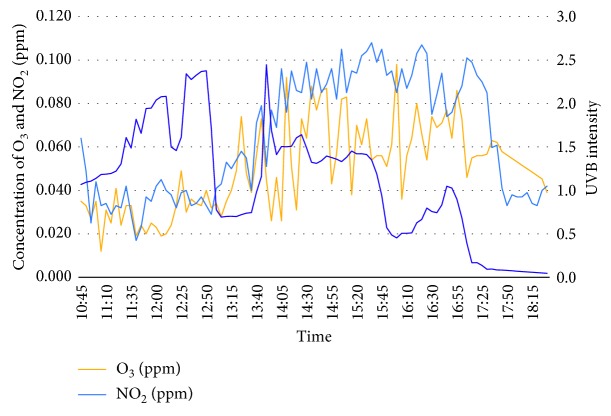
Concentration of O_3_, NO_2_, and UVB Intensity for day 2 (23^rd^ Feb 2018).

On day 2, which is on the 23^rd^ February 2018, the surrounding temperature fluctuated when approaching peak hours in the evening while relative humidity is seen to be stabilized from morning till midday with a slight growth during peak hours. Concentration of ozone and nitrogen dioxide has shown almost the same pattern as the first day. However, UVB intensity fluctuated during midday probably from cloud coverage, and this is proven when the surrounding temperature felt to below 32°C at similar time.  Figure 4 shows the graph of concentration of ozone, nitrogen dioxide, and UVB intensity.

**Figure 5:**
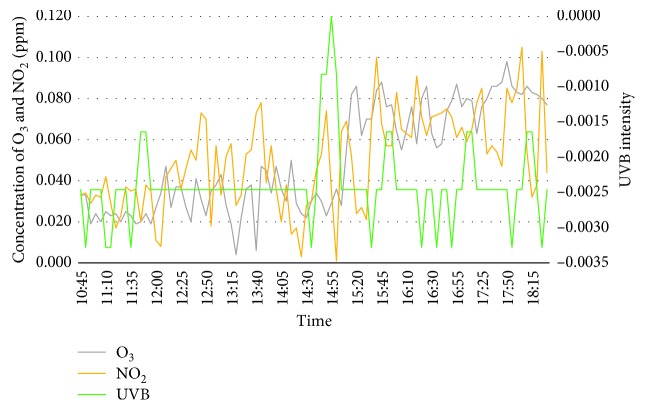
Concentration of O_3_, NO_2_, and UVB intensity for day 3 (8^th^ March 2018).

8^th^ March 2018 has been selected as day 3 to run the experiment. The temperature has exceeded 35°C during midday, and around 60% of relative humidity is observed at the same period. Concentration of ozone felt significantly from 1 to 2 pm as the wind started. Otherwise, stable concentration trend is seen from morning till late afternoon before it spiked up at 3 pm. It then continued to rise but gradually felt approaching evening. On the other hand, the concentration has shown inconsistent pattern compared to the ozone. It has dropped to almost zero due to the windy condition but a steady growth can be seen during peak hours because of congested vehicles. Even though the temperature is the highest compared to the previous experiment, the cloud coverage has prevented the UVB rays to reach the troposphere layer of the Earth, and this can be explained by the negative value of UVB intensity from the sensor. Therefore, UVB intensity did not play aid in ozone formation during day 3.  Figure 5 shows the graph of concentration of ozone, nitrogen dioxide, and UVB intensity.

**Figure 6:**
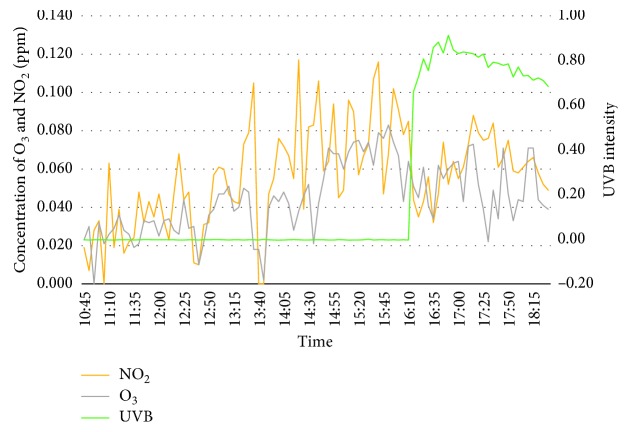
Concentration of O_3_, NO_2_, and UVB intensity for day 4 (19^th^ March 2018).

The fourth day of the experiment is on 19^th^ March 2018. The surrounding temperature was the highest compared to the previous days of experiment that was above 40°C. However, relative humidity was the lowest during that day. Both concentration of ozone and nitrogen dioxide felt sharply at one point during midday. This happened due to the wind speed from vehicles passing by. The increase and decline of concentration for both ozone and nitrogen dioxide showed the same trend at the same time. The negative value of UVB intensity is due to the cloud coverage similar to the third day of the experiment.  Figure 6 shows the graph of concentration of ozone, nitrogen dioxide, and UVB intensity.

**Figure 7:**
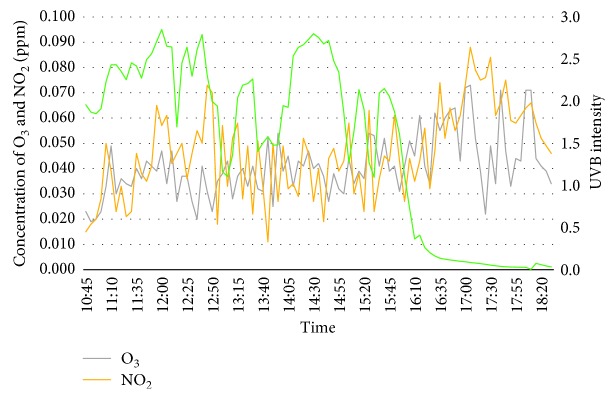
Concentration of O_3_, NO_2_, and UVB intensity for day 5 (24^th^ April 2018).

Day 5 of experiment has recorded the surrounding temperature between 30 and 35°C, with the highest peak during midday. Concentration of ozone and nitrogen dioxide has been seen with similar trend throughout the day. Sharp fluctuation could be from the wind and the vehicles passing by while the concentration rose to above the pollution limit during peak hour due to congest traffic condition along the road to exit and enter Bangi. It slowly decreases from 6 pm onwards as the traffic continue to sail smoothly. On the other hand, UVB intensity experienced a significant rise and fell throughout the day with the highest value recorded in the morning and midday and slowly decline from the late afternoon till 6 pm.  Figure 7 shows the graph of concentration of ozone, nitrogen dioxide, and UVB intensity for the fifth day of experiment.

**Figure 8:**
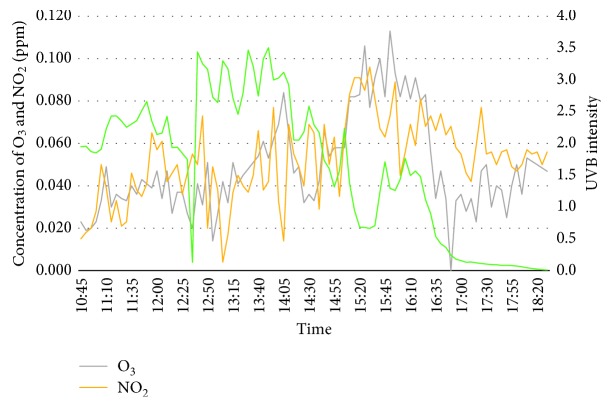
Concentration of O_3_, NO_2_, and UVB intensity for day 6 (27^th^ April 2018).

On the sixth day of experiment, the surrounding temperature is also high and has approaching 40°C during afternoon but it is rather cloudy throughout the day. The UVB intensity is recorded at value less than 5 mW/cm^2^ and slowly declining from midday to the evening. During the peak hour, the intensity is approaching zero value, and at this time, the cloud covered most of the sunlight rays. Both ozone and nitrogen dioxide recorded the same trend but ozone experienced a significant fell to almost zero before the peak hours.  Figure 8 shows the graph of concentration of ozone, nitrogen dioxide, and UVB intensity on the sixth day of experiment.

**Figure 9:**
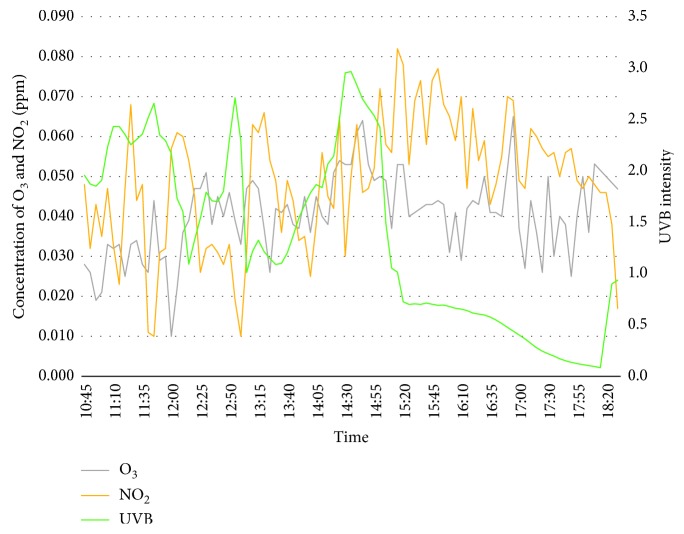
Concentration of O_3_, NO_2_, and UVB intensity for day 7 (30^th^ April 2018).

The final day of experiment (30^th^ April 2018) shows that the surrounding weather is behaving in similar condition from the sixth day of experiment, with high temperature and high relative humidity due to the cloudy condition throughout the whole day. Concentration of ozone rose steadily from midday till peak hour at 6 pm and continues to decrease slowly as the traffic jam flow smoothly afterwards. Due to cloudy and windy days, the ozone concentration did not reach the pollution limit with the same traffic compared to the previous days of experiment. Also, the UVB intensity during peak hours slowly declines as the weather gets very cloudy. Concentration of nitrogen dioxide however did not experience the same trend as ozone because the amount of traffic is still the same as the previous days.  Figure 9 shows the graph of concentration of ozone, nitrogen dioxide, and UVB intensity for the seventh day of experiment.

We have chosen 7 hours (daytime) as the monitoring time due to the presence of sunlight, UVA, and UVB during the day. Ozone is formed with the presence of sunlight, UVA and UVB, and also carbon monoxide. During midday from 11 am until 2.30 pm (Malaysia time), traffic is congested, which in turn produce a lot of carbon monoxide from carbon fuels generated vehicles. Carbon monoxide reacts with sunlight and UVA/UVB to form ozone. Thus, it is proven that, during congestion, abundance of nitrogen oxide and volatile organic compound (VOC) in the environment will catalyze the formation of ozone.

However, in the early morning, traffic is not as bad as during midday and evening. Thus, the monitoring time starts at 11 am until 6 pm. The data are then analyzed using the IBM SPSS with Pearson correlation chosen as the correlation type. Concentration of ozone and its relationship with other parameters is determined, and we can see which parameter and factor plays an important role in the formation of ozone.  Table 2 shows the Pearson correlation from day 1 to day 7 of experiment. In addition, by applying similar software, we can also obtain the linear regression and its graph to see the relationship between the parameter graphically.

Based on the result obtained from all 7 days of experiment, it is obvious that the concentration of ozone has reached above the permitted limit. Safety measures have to be taken to prevent this situation from becoming worst and will risk our health and environment. From the data analysis, we can see that the concentration of nitrogen dioxide plays an important role for the formation of ozone. Other factors such as UV intensity also give an impact especially for UVB intensity.

**Figure 10:**
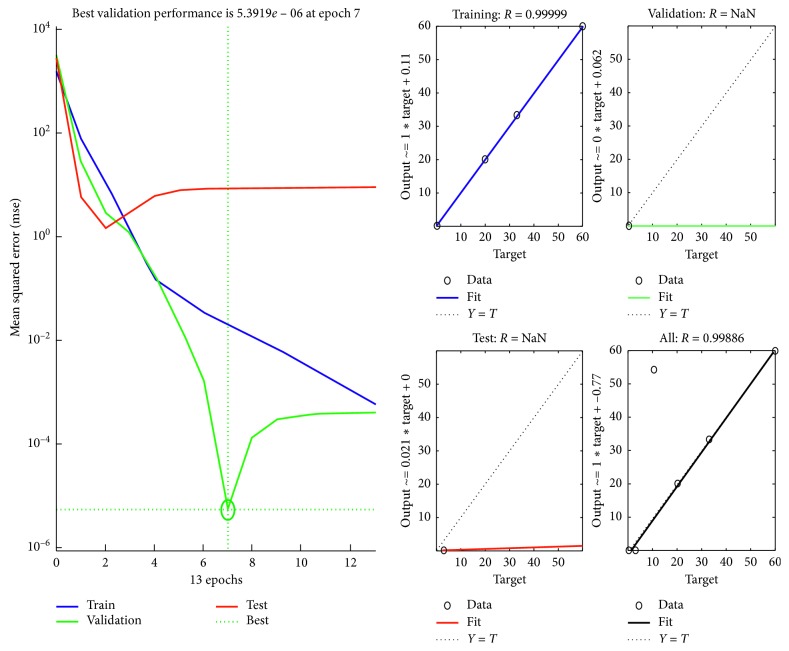
Performance and regression plot for day 2.

**Figure 11:**
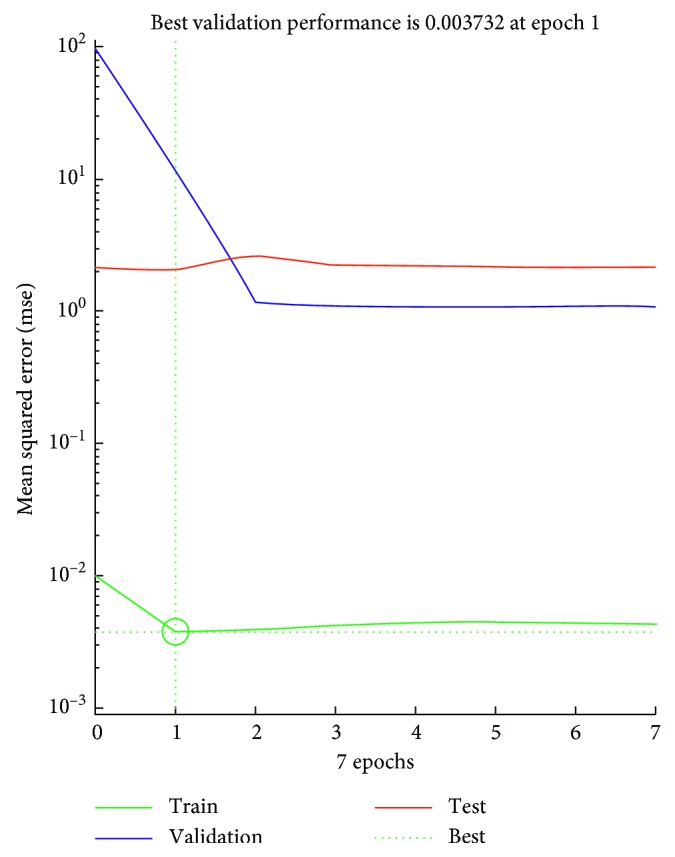
Performance plot for day 3 based on forecasted data of day 2.

Later, ANN is applied to optimize the MSE and provide a reliable forecast on the next day of the ozone concentration. The successfulness of developed ANN models are evaluated by comparing its predicted output results, which in this case is the next day forecasted pollution based on the training network, validation, and testing data sets [16]. Regression value varying from 0 to 100 percent shows the performance of the model. Forward stepwise selection identifies that concentration of nitrogen dioxide gave the significance effect while eliminating RH as the least significant impact. Therefore, the optimized network with all inputs of the experimental results versus the predicted output is plotted and presented in Figure 10 for day 2, and based on the forecasted results, a new plot is generated for day 3, as given in Figure 11.

**Figure 12:**
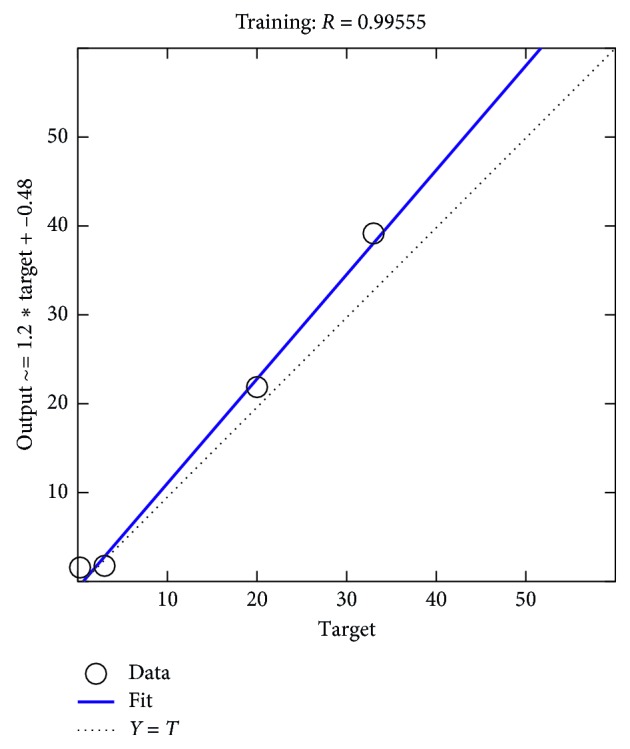
Performance plot for day 3 on NO_2_ concentration.

**Figure 13:**
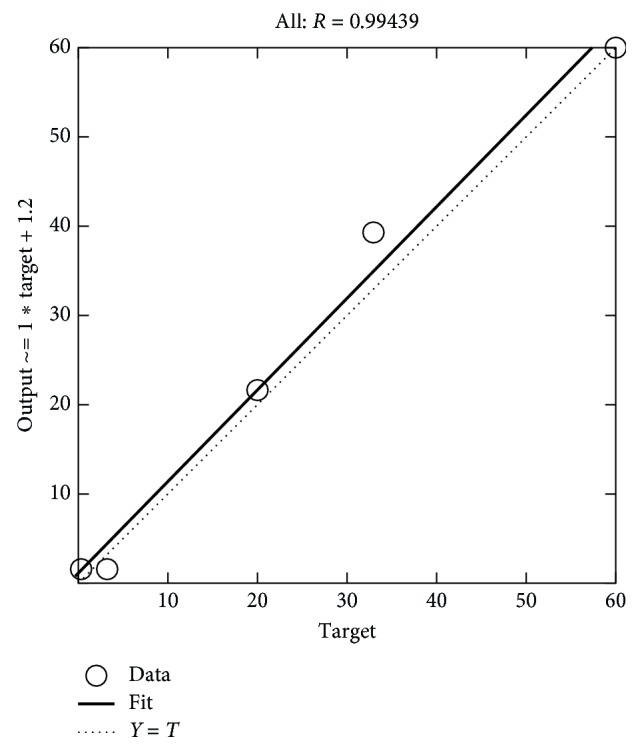
Performance plot for day 3 on UVB rays.

The optimized MSE shows the value less than 0.01 for day 3, which is the tested data set. The output performance is given in Figure 12 for concentration of NO_2_ and Figure 13 for UVB rays. Based on the results, it can be concluded that an ANN model performs better in forecasting the ozone concentration pattern and is comparable with the experimental data.

## 4. Conclusion

The study concludes that, by choosing inputs to represent hourly emission patterns and its relationship to atmospheric temperature, relative humidity, and solar radiation (UVA and UVB), the simple ANN model can give a reliable forecast of zone concentration for the next day. The predictor model uses the variable that is obtainable from routine monitoring and data recorded. When the most influencing factors have been identified through the sensitivity analysis, a single network can be used for multiple pollutants based on the common influencing parameters. Meteorology condition and emission patterns play an important part in influencing the ozone concentration.

## Figures and Tables

**Table 1:** Detail description of the ANN framework.

Specification	Description
Toolbox	Artificial neural network
Parameter tested	(i) Temperature
	(ii) Relative humidity (%RH)
	(iii) Concentration of nitrogen dioxide
	(iv) UVA rays
	(v) UVB rays
Type of network	Feed-forward back propagation/perceptron
Input layer	Data from previous day (i.e., day 1)
Output layer	The forecasted data based on input data (i.e., day 2)
Targetted range	(i) Ozone: 0.06 ppm
	(ii) NO_2_: 0.04 ppm
	(iii) Temperature, %RH, UVA, and UVB not specified
Neuron	10 layer

**Table 2:** Pearson correlation for concentration of ozone.

Parameter	Temperature	Relative humidity	NO_2_	UVA	UVB
Day 1	0.334	0.212	0.458	−0.364	−0.457
Day 2	0.167	0.046	0.628	−0.058	−0.325
Day 3	0.313	−0.363	0.480	−0.398	−0.058
Day 4	−0.069	−0.089	0.553	−0.380	−0.224
Day 5	0.560	−0.446	0.349	−0.467	−0.470
Day 6	0.145	−0.048	0.477	−0.019	−0.017
Day 7	0.189	−0.382	0.165	−0.034	−0.060

## Data Availability

The experimental data used to support the findings of this study are currently under embargo while the research findings are commercialized. Requests for data, 12 months after publication of this article, will be considered by the corresponding author.
